# Diagnostic Yield of Liver Biopsy in Porto-Sinusoidal Vascular Disorder: Are Conventional Adequacy Criteria Always Indispensable?

**DOI:** 10.7759/cureus.111053

**Published:** 2026-06-17

**Authors:** Ikram Aluahabi, Mohamed Borahma, Nawal Lagdali, Fatima Zahra Chabib, Sabbah Selma, Fatima Zohra Ajana, Maryeme Kadiri

**Affiliations:** 1 Hepatogastroenterology C, Ibn Sina University Hospital, Mohammed V University, Rabat, MAR

**Keywords:** adequacy criteria, histology, liver biopsy, portal hypertension, porto-sinusoidal vascular disorder

## Abstract

Background and aims: Porto-sinusoidal vascular disorder (PSVD) is a rare vascular liver disease defined by the absence of cirrhosis on a liver biopsy together with specific histological lesions and/or characteristic signs of portal hypertension. Although current recommendations define an adequate biopsy as a specimen of at least 20 mm in cumulative length, containing at least 10 portal tracts, and showing minimal fragmentation, the real-world diagnostic yield of biopsies that fail to meet all these criteria remains insufficiently explored. We aimed to evaluate the contribution of liver biopsy to PSVD diagnosis and to determine whether strict fulfillment of conventional adequacy criteria is indispensable for detecting diagnostic histological lesions.

Methods: We conducted a retrospective, descriptive, and analytical study over a nine-year period (2017-2026) in a tertiary care hepatogastroenterology department. All consecutive patients diagnosed with PSVD who underwent liver biopsy were included. Clinical, endoscopic, radiological, etiological, therapeutic, and histological data were collected and analyzed. Patients diagnosed on histological criteria were compared with those diagnosed on portal hypertension criteria alone. Factors associated with positive histology were evaluated using univariate and multivariate logistic regression analyses.

Results: Forty-three patients were included (mean age 42.4±12.6 years; male-to-female ratio, 0.79). The most common clinical presentations were anemic syndrome, 17 (39.5%); abdominal pain, 14 (32.6%); and hemorrhagic presentation, 12 (27.9%). Upper endoscopy demonstrated esophageal varices in 31 (72.1%) patients, gastroesophageal varices in eight (18.6%), and portal hypertensive gastropathy in nine (20.9%). Portal vein thrombosis was documented in 11 (25.6%) patients and portal cavernoma in four (9.3%). Protein C and/or S deficiency was the most frequent associated condition, occurring in 14 (32.6%) patients. Histological criteria supported the diagnosis in 26 (60.5%) patients, including 17 (39.5%) with at least one specific histological lesion. Only two biopsies (4.7%) fulfilled all conventional adequacy criteria. Nevertheless, diagnostic histological lesions were identified in a substantial proportion of biopsies that did not meet all criteria. In both univariate and multivariate analyses, cumulative biopsy length, number of portal tracts, and number of cores were not independently associated with positive histology.

Conclusion: Liver biopsy remained a cornerstone of PSVD diagnosis in this cohort. However, diagnostic histological lesions were frequently identified in biopsies not meeting all conventional adequacy criteria, and biopsy-related quantitative parameters were not predictive of diagnostic yield. These findings suggest that diagnostic histological information may still be obtained from suboptimal biopsy specimens. No association was detected in this cohort between the assessed biopsy adequacy parameters and positive histology, but these results should be interpreted cautiously in light of the study design and sample size. Larger multicenter studies are warranted to establish evidence-based adequacy thresholds specific to PSVD.

## Introduction

Porto-sinusoidal vascular disorder (PSVD) is a rare vascular liver disease defined by the absence of cirrhosis on liver biopsy in combination with specific histological lesions and/or signs of portal hypertension. Since its formal proposal by the Vascular Liver Disease Interest (VALDIG) group in 2019, the concept of PSVD has unified several previously fragmented clinicopathological entities, including obliterative portal venopathy, nodular regenerative hyperplasia, hepatoportal sclerosis, and incomplete septal fibrosis/cirrhosis, under a single diagnostic framework centered on small portal venous and sinusoidal lesions [1].

Despite growing recognition, PSVD remains challenging to diagnose in everyday clinical practice. Its clinical presentation is heterogeneous, ranging from incidentally discovered liver enzyme abnormalities to variceal bleeding, portal vein thrombosis, and other complications of portal hypertension. Imaging and liver stiffness measurements may raise clinical suspicion, but liver biopsy remains central to the diagnostic workup because it is required to exclude cirrhosis and, when possible, identify specific vascular lesions [1-6].

Biopsy quality is therefore a major practical concern. Classical recommendations have generally defined an adequate specimen as one measuring at least 20 mm in cumulative length, containing at least 10 portal tracts, and displaying little or no fragmentation, or otherwise deemed interpretable by an experienced liver pathologist [2-4]. In real-world practice, however, many biopsies fail to meet all these criteria because of fragmentation, limited length, or a low portal tract count. Whether such specimens should be considered non-diagnostic in suspected PSVD remains unresolved.

The present study aimed to describe the clinical, etiological, and histological profile of patients with PSVD managed at a Moroccan tertiary referral center and to assess the diagnostic yield of liver biopsy for identifying histological lesions compatible with PSVD in a cohort composed predominantly of suboptimal biopsy specimens.

## Materials and methods

Study design and patients

This retrospective, descriptive, and analytical study was conducted in the hepatogastroenterology department of a tertiary referral center over a nine-year period from January 2017 to May 2026. All consecutive patients diagnosed with PSVD who underwent liver biopsy during the study period were eligible for inclusion. Diagnosis was established according to contemporary clinicopathological criteria following exclusion of cirrhosis and major competing causes of portal hypertension incompatible with PSVD. According to VALDIG criteria, PSVD was diagnosed in the absence of cirrhosis when at least one specific histological sign or specific sign of portal hypertension was present, or when at least one non-specific histological sign was associated with at least one non-specific sign of portal hypertension [1-4]. Specific histological signs included obliterative portal venopathy, nodular regenerative hyperplasia, and incomplete septal fibrosis/cirrhosis. Non-specific histological signs included portal tract abnormalities, irregular distribution of portal tracts and central veins, non-zonal sinusoidal dilatation, and mild perisinusoidal fibrosis. Specific signs of portal hypertension included gastroesophageal or ectopic varices, portal hypertensive bleeding, and portosystemic collaterals on imaging. Non-specific signs of portal hypertension included ascites, thrombocytopenia, and splenomegaly. All liver biopsies were performed percutaneously using a 16-gauge needle, with one to two passes depending on specimen quality and operator assessment. The study was conducted in accordance with the Declaration of Helsinki and local institutional ethics requirements.

Data collection

Data were extracted from the institutional database and individual medical records. The following variables were collected: age at diagnosis, sex, clinical presentation, endoscopic findings, vascular imaging features, associated conditions, therapeutic management, and clinical outcomes. Biopsy-related variables included cumulative specimen length, adequacy of specimen length, number of portal tracts, adequacy of portal tract number, fragmentation status, and number of cores.

For the purposes of this study, a biopsy was defined as fully adequate according to conventional criteria when it measured at least 20 mm in cumulative length, contained at least 10 portal tracts, and showed minimal fragmentation [2-4]. In fragmented biopsies, specimen length referred to the cumulative length of all fragments. Histological lesions were categorized as specific or non-specific for PSVD. For the purposes of the present analysis, histology was considered positive when the biopsy showed either specific or non-specific histological signs compatible with PSVD.

Statistical analysis

The primary objective was to assess the diagnostic yield of liver biopsy for identifying histological lesions compatible with PSVD in predominantly suboptimal biopsy specimens. Patients diagnosed on histological criteria were compared with those diagnosed on portal hypertension criteria alone. Factors potentially associated with positive histology were analyzed using univariate and multivariate logistic regression models; covariates included adequate specimen length, adequate number of portal tracts, and specimen fragmentation. Continuous variables are expressed as mean ± standard deviation and categorical variables as frequencies and percentages. Given the limited sample size, the study had restricted statistical power, particularly for multivariable analysis, and negative findings were therefore interpreted with caution. A two-sided p-value of less than 0.05 was considered statistically significant. All analyses were performed using Jamovi.

## Results

Baseline characteristics

The study included 43 patients with a mean age at diagnosis of 42.4±12.6 years. The cohort comprised 19 (44%) men and 24 (56%) women, yielding a male-to-female ratio of 0.79. Clinical presentation was dominated by anemic syndrome in 17 (39.5%) patients, abdominal pain in 14 (32.6%), and hemorrhagic presentation in 12 (27.9%). The most common associated condition was protein C and/or S deficiency, identified in 14 (32.6%) patients. Other associated conditions included myelofibrosis, Biermer disease, Behçet disease, celiac disease, factor V (Leiden) mutation, and paroxysmal nocturnal hemoglobinuria. Baseline clinical, endoscopic, vascular, etiological, and therapeutic characteristics are summarized in Table 1. Upper gastrointestinal endoscopy revealed esophageal varices in 31 (72.1%) patients, gastroesophageal varices in eight (18.6%), and portal hypertensive gastropathy in nine (20.9%). Splenomegaly exceeding 13 cm was present in 35 (81.4%) patients on imaging. Portal vein thrombosis was documented in 11 (25.6%) patients and portal cavernoma in four (9.3%). Beta-blockers were prescribed in 29 (67.4%) patients, and anticoagulation was initiated in 16 (37.2%), predominantly with rivaroxaban in 11 (68.8%).

**Table 1:** Baseline clinical, endoscopic, vascular, etiological, and therapeutic characteristics of the study population (n=43)

Variable	n (%)
Clinical presentation	
Hemorrhagic presentation	12 (27.9)
Abdominal pain	14 (32.6)
Anemic syndrome	17 (39.5)
Endoscopic findings	
Esophageal varices	31 (72.1)
Gastroesophageal varices	8 (18.6)
Portal hypertensive gastropathy	9 (20.9)
Vascular imaging findings	
Portal vein thrombosis	11 (25.6)
Portal cavernoma	4 (9.3)
Splenomegaly (>13 cm)	35 (81.4)
Associated conditions	
Protein C and/or S deficiency	14 (32.6)
Myelofibrosis	3 (7.0)
Biermer disease	2 (4.7)
Behçet disease	2 (4.7)
Celiac disease	2 (4.7)
Factor V (Leiden) mutation	2 (4.7)
Paroxysmal nocturnal hemoglobinuria	1 (2.3)
Treatment	
Beta-blocker therapy	29 (67.4)
Anticoagulation	16 (37.2)

Liver biopsy characteristics and histological yield

Histological criteria directly supported the diagnosis of PSVD in 26 (60.5%) of 43 patients. Of these, 17 (39.5%) had at least one specific histological lesion, whereas nine (20.9%) had only non-specific features. Among specific lesions, obliterative portal venopathy was the most frequent finding, identified in 10 (23.3%) patients, followed by incomplete septal fibrosis/cirrhosis in four (9.3%). Nodular regenerative hyperplasia was not explicitly documented in any biopsy report. Only two biopsies (4.7%) fulfilled all conventional literature-based adequacy criteria.

Nevertheless, diagnostic histological lesions were identified in many biopsies that were shorter than the recommended threshold, fragmented, or contained fewer than 10 portal tracts. This discrepancy between formal adequacy and actual histological yield constitutes the central finding of the present study. Biopsy adequacy data and histological findings are detailed in Table 2.

**Table 2:** Liver biopsy adequacy profile and histological findings (n=43) PTs: portal tracts

Variable	n (%)
Biopsy adequacy	
Biopsies fully adequate by conventional criteria (≥20 mm, ≥10 PTs, minimal fragmentation)	2 (4.7)
Biopsies not fully adequate by conventional criteria	41 (95.3)
Diagnostic yield	
Diagnosis supported by histological criteria	26 (60.5)
Specific histological lesion(s) identified	17 (39.5)
Non-specific histological lesions only	9 (20.9)
Diagnosis based on portal hypertension criteria only	17 (39.5)
Specific histological lesions	
Obliterative portal venopathy	10 (23.3)
Incomplete septal fibrosis/cirrhosis	4 (9.3)
Nodular regenerative hyperplasia	0 (0.0)

Adequate specimen length alone was observed in 11 (25.6%) patients, but all of these samples were fragmented, while an adequate number of portal tracts was found in six (14%) patients. Biopsy specimens were fragmented in 18 (41.9%) patients. Comparison of patients diagnosed based on specific and non-specific histological criteria with those diagnosed based on specific signs of portal hypertension revealed no significant differences between the two groups in terms of core size and number of portal tracts (Table 3).

**Table 3:** Comparison of diagnoses based on histological findings and specific signs of portal hypertension

	Histological criteria (n=26)	Portal hypertension criteria alone (n=17)	P
Adequate core size	8/26 (30.8%)	3/17 (17.6%)	0.480
Adequate number of portal spaces	4/26 (15.4%)	2/17 (11.8%)	1.000

Factors associated with positive histology

In both univariate and multivariate analyses, none of the assessed biopsy adequacy parameters, including adequate specimen length, adequate number of portal tracts, and specimen fragmentation, was independently associated with the presence of diagnostic histological lesions of PSVD (Table 4). The odds ratios were close to unity, with wide confidence intervals and non-significant p-values in both analytical models.

**Table 4:** Logistic regression analysis of factors associated with positive histology OR: odds ratio; CI: confidence interval

Variable	Univariate OR	95% CI	P	Multivariate OR	95% CI	P
Adequate specimen length	1.09	0.88-1.37	0.426	1.12	0.86-1.47	0.398
Adequate number of portal tracts	1.01	0.79-1.30	0.935	0.97	0.73-1.28	0.827
Fragmented specimen	0.87	0.43-1.77	0.700	1.03	0.42-2.56	0.941

## Discussion

Definition and conceptual framework

The term PSVD was proposed by the VALDIG group in 2019 to unify several previously used clinicopathological labels, including obliterative portal venopathy, nodular regenerative hyperplasia, hepatoportal sclerosis, and incomplete septal fibrosis/cirrhosis, under a single framework centered on small portal venous and sinusoidal lesions [1,7]. According to this definition, diagnosis requires a liver biopsy excluding cirrhosis together with at least one of the following: a specific sign of portal hypertension, a specific histological lesion, or the combination of one non-specific portal hypertension sign and one non-specific histological feature [1,2]. The 2025 European Association for the Study of the Liver (EASL) Clinical Practice Guidelines on vascular liver diseases maintained this biopsy-based approach and emphasized that PSVD may coexist with other chronic liver diseases and with portal vein thrombosis, provided cirrhosis and other exclusion diagnoses are carefully ruled out [8]. Our cohort was assembled according to this contemporary VALDIG/EASL framework, which strengthens the applicability of our findings to current clinical practice.

Epidemiology

PSVD remains uncommon and is likely under-recognized in clinical practice, particularly among patients initially labeled as having cryptogenic cirrhosis or unexplained portal hypertension [3-5,7]. Published cohorts consistently report diagnosis in the fourth to fifth decade, with geographic variation in sex distribution: male predominance is described in many Western and Indian cohorts, whereas female predominance is reported in Japanese series [1,3-5]. In the recent multicenter study by Magaz et al., including 587 patients with PSVD and portal hypertension, the median age was 47 years and 38% were women [9]. Our cohort appears slightly younger (mean age 42.4 years) with a moderate female predominance (55.8%), findings that remain within the epidemiological spectrum reported in the literature and may partly reflect regional patient characteristics.

Associated conditions

PSVD is frequently associated with prothrombotic, immunological, hematological, infectious, genetic, and drug-related conditions, rather than being truly idiopathic in every case. The 2025 EASL Clinical Practice Guidelines recommend a systematic search for these associated conditions at the time of initial diagnosis, encompassing thrombophilia, immune-mediated disorders, hematological diseases, HIV infection, prior exposure to thiopurines or oxaliplatin, and selected genetic contexts [8]. Recent data also suggest that PSVD with known associated etiologies may present with more severe portal hypertension, poorer liver function reserve, higher prevalence of portal vein thrombosis, and worse outcomes than apparently idiopathic forms [10]. In our cohort, protein C and/or S deficiency was the most frequent associated condition (32.6%), followed by myelofibrosis, Biermer disease, Behçet disease, celiac disease, factor V (Leiden) mutation, and paroxysmal nocturnal hemoglobinuria, a distribution consistent with the predominance of prothrombotic and immune-mediated conditions reported in the literature.

Liver biopsy adequacy and diagnostic criteria

The cornerstone of PSVD diagnosis is the confident exclusion of cirrhosis combined with the identification of vascular-type histological lesions [1-4,8]. Historically, an adequate biopsy has been defined as a specimen measuring at least 20 mm, containing at least 10 portal tracts, and showing little or no fragmentation, or deemed adequate by an experienced liver pathologist [1-4]. However, this threshold was largely consensus-based rather than empirically validated in the specific context of PSVD. In addition, liver biopsy has shown substantial diagnostic value in the etiological workup of unexplained portal hypertension, with PSVD representing an important non-cirrhotic cause that should not be overlooked [11]. More recently, de Broucker et al. demonstrated that, in suspected PSVD, a 15 mm biopsy with at least one 10 mm fragment was sufficient to exclude cirrhosis with high confidence, suggesting that traditional adequacy criteria may be overly restrictive for routine practice [12]. Our study extends this observation by showing that diagnostic histological lesions were identified in a substantial proportion of biopsies that did not fulfill all of these criteria and that biopsy-related quantitative parameters were not predictive of histological yield in multivariate analysis.

Clinical and paraclinical features

PSVD frequently remains silent until portal hypertension becomes clinically overt; when symptoms occur, variceal bleeding represents one of the classic revealing events, while abdominal pain and manifestations related to hypersplenism are also commonly observed [2-5,9,13]. In our cohort, hemorrhagic presentation accounted for 12 (27.9%) of cases, abdominal pain for 14 (32.6%), and anemic syndrome for 17 (39.5%), confirming that patients in this setting are often diagnosed after clinically meaningful portal hypertension has already developed. Upper endoscopy revealed esophageal varices in 31 (72.1%) of patients, a proportion consistent with reported rates in large contemporary series [2,3,9].

Biological abnormalities in PSVD are typically modest compared with cirrhosis, as the disease is characterized by relatively preserved synthetic liver function despite established portal hypertension [2-5,11]. Thrombocytopenia is one of the most common laboratory clues, while transaminases, cholestatic enzymes, and bilirubin may be normal or only mildly elevated [2-5,9]. Splenomegaly exceeding 13 cm was present in 35 (81.4%) of our patients on abdominal imaging, and portal vein thrombosis was documented in 11 (25.6%), reflecting the recognized vascular phenotype of PSVD and the central role of imaging in the diagnostic workup [3-5,8].

Histological features

Histology remains the defining element of PSVD. Specific lesions include obliterative portal venopathy (also referred to as portal vein stenosis or obliteration), nodular regenerative hyperplasia, and incomplete septal fibrosis/cirrhosis, whereas non-specific lesions encompass portal tract abnormalities, irregular distribution of portal tracts and central veins, non-zonal sinusoidal dilatation, and mild perisinusoidal fibrosis [1-5,14,15]. These lesions may be subtle, patchy, and heterogeneously distributed across the biopsy specimen, which explains why sampling limitations are a central concern and why the reporting pathologist must actively search for both specific and supportive patterns rather than rely on a single pathognomonic feature [3,12,15]. In our study, specific histological signs were found in 17 (39.5%) of biopsies and non-specific signs in nine (20.9%), while biopsy quantitative metrics were not significantly associated with positive histology, a finding compatible with the uneven topography of PSVD lesions.

Management

There is currently no PSVD-specific disease-modifying therapy; management is therefore based on the treatment of portal hypertension complications and associated conditions [2-5,8,9,13]. Current recommendations support variceal management following principles analogous to those applied in cirrhosis, including non-selective beta-blockers and endoscopic prophylaxis, while anticoagulation is individualized based on the presence of portal vein thrombosis and the underlying thrombophilic risk [2,3,8]. Transjugular intrahepatic portosystemic shunt and liver transplantation remain options in selected severe cases [2,3,8,9]. In our cohort, beta-blockers were prescribed in 29 (67.4%) of patients and anticoagulation in 16 (37.2%), a distribution coherent with both the high endoscopic burden of portal hypertension and the frequent thrombotic or prothrombotic background.

Strengths and limitations

The main limitations of the present study are its retrospective single-center design, the relatively small sample size, and the limited statistical power of the analysis. In particular, only 43 patients were included and only two biopsy specimens fulfilled all conventional adequacy criteria, which precluded a formal comparison between fully adequate and non-fully adequate biopsies. Accordingly, this study should be interpreted primarily as an assessment of diagnostic yield in predominantly suboptimal biopsy specimens. In addition, because only patients already diagnosed with PSVD were included, selection bias cannot be excluded and the study does not allow assessment of the impact of suboptimal biopsies on diagnostic sensitivity in an unselected population with suspected PSVD. The multivariable analysis should also be interpreted with caution because the low number of events may have limited the ability to detect modest associations and may increase the risk of model overfitting. Another limitation is the retrospective pathology assessment, which may have led to under-recording of subtle histological lesions and limited standardization of histological review. Nevertheless, this study provides one of the few North African series specifically addressing liver biopsy adequacy in the context of PSVD. Its strengths include the use of contemporary VALDIG/EASL diagnostic criteria and the systematic collection of biopsy-related parameters in a clinically relevant and underrepresented setting.

## Conclusions

In this single-center cohort, liver biopsy remained essential for PSVD diagnosis, and diagnostic histological lesions were frequently identified in biopsy specimens that did not fulfill all conventional adequacy criteria. However, no association was detected in this cohort between the assessed biopsy adequacy parameters and diagnostic histological yield. These results should be interpreted cautiously in light of the retrospective design, limited sample size, and low number of fully adequate biopsies. Our findings suggest preserved diagnostic yield in predominantly suboptimal specimens but do not justify relaxing current adequacy standards. Larger multicenter prospective studies are needed to clarify this issue.
